# Oxidative Stress and Antioxidants in the Diagnosis and Therapy of Periodontitis

**DOI:** 10.3389/fphys.2017.01055

**Published:** 2017-12-14

**Authors:** L'ubomíra Tóthová, Peter Celec

**Affiliations:** ^1^Faculty of Medicine, Institute of Molecular Biomedicine, Comenius University, Bratislava, Slovakia; ^2^Faculty of Medicine, Institute of Physiology, Comenius University, Bratislava, Slovakia; ^3^Faculty of Medicine, Institute of Pathophysiology, Comenius University, Bratislava, Slovakia; ^4^Department of Molecular Biology, Faculty of Natural Sciences, Comenius University, Bratislava, Slovakia

**Keywords:** reactive oxygen species, free radicals, antioxidative therapy, systematic review, oral diseases

## Abstract

Oxidative stress has been implicated in the pathogenesis of numerous diseases. However, large interventional studies with antioxidants failed to show benefits in the prevention or treatment of cardiovascular diseases, cancer, or diabetes mellitus. Numerous clinical studies have confirmed the association of oxidative stress markers and periodontitis. Technical and biological variability is high for most of the analyzed markers and none of them seems to be optimal for routine clinical use. In a research setting, analysis of a palette of oxidative stress markers is needed to cover lipid peroxidation, protein oxidation, and the antioxidant status. The source of reactive oxygen species and their role in the pathogenesis of periodontitis remains unclear. Interventional experiments indicate that oxidative stress might be more than just a simple consequence of the inflammation. Small studies have confirmed that some antioxidants could have therapeutic value at least as an addition to the standard non-surgical treatment of periodontitis. A clear evidence for the efficiency of antioxidant treatment in large patient cohorts is lacking. Potentially, because lowering of oxidative stress markers might be a secondary effect of anti-inflammatory or antibacterial agents. As the field of research of oxidative stress in periodontitis gains attraction and the number of relevant published papers is increasing a systematic overview of the conducted observational and interventional studies is needed. This review summarizes the currently available literature linking oxidative stress and periodontitis and points toward the potential of adjuvant antioxidant treatment, especially in cases where standard treatment fails to improve the periodontal status.

## Introduction

Oxidative stress is both, a pathomechanism involved in numerous inflammatory diseases causing damage to lipids, nucleic acids and proteins—oxidative distress, as well as an important physiological process that enables the immune system to cope with microorganisms and intracellular cell signaling—oxidative eustress (Sies et al., 2017). Which edge of the sword is the dominant depends on the delicate balance between the production of reactive oxygen/nitrogen species and the antioxidant capacity of the tissue. The physiological functions of free radicals have been neglected for years and so, much more is known about the pathological role of oxidative stress. A variety of free radicals is produced and interacts with a variety of substrates. This leads to a palette of biomarkers that can be used for the assessment of oxidative stress-induced damage (Frijhoff et al., 2015).

Oxidative stress is usually defined as a disbalance of the production of free radicals and antioxidant mechanisms (Kopáni et al., 2006). However, free radicals are not a simple negative byproduct of oxygen metabolism. They are involved in immune responses, liver metabolism, but also in intracellular signaling pathways (Espinosa-Diez et al., 2015). The mechanisms involved in the physiological intracellular role of free radicals include the modulation of cysteine residues of redox-sensitive enzymes and other regulatory proteins (Finkel, 2011; Russell and Cotter, 2015). It has been hypothesized that under physiological conditions even high concentrations of any one primary reactive oxygen or nitrogen species does not lead to oxidative damage, as the cell has preventive and reparative tools to cope with the radical. The reactions of superoxide, nitric oxide, and other primary reactive species are reversible and are ideal for intracellular signaling (Weidinger and Kozlov, 2015). Thus, measuring oxidative stress using any one marker can lead to wrong interpretations. This might include the hope that administration of antioxidants will effectively treat diseases associated with oxidative stress. As recently hypothesized, one of the prototypic oxidative stress diseases—diabetes mellitus might be actually a consequence of deficiency of reactive oxygen species rather than of oxidative damage (Watson, 2014). It should not escape our notice that this proposed mechanism might suggest that antioxidants could increase the risk rather than prevent metabolic diseases.

Periodontitis is an inflammatory disease affecting supporting structures of the teeth leading at the end to loss of alveolar bone and teeth (Kinane et al., 2017). The main causative factor are microorganisms that colonize the subgingival dental plaque inducing an inflammatory host response. The inflammation affects, however, also the surrounding healthy tissue ultimately leading to the destruction of the periodontium (Kinane et al., 2011). Although lipopolysaccharide and proteolytic enzymes are essential in periodontitis, exaggerated inflammatory response, genetic predisposition, smoking, bad oral hygiene, and malnutrition are important in pathogenesis of periodontitis as well (Laine et al., 2012).

The role of oxidative stress in periodontitis has been postulated already decades ago (Shapira et al., 1991; Chapple, 1997). However, the suggested involvement was not clear. Some studies showed that leukocytes from patients with periodontitis are exhausted and have a low oxidation activity (Loesche et al., 1988), other studies pointed toward higher production of free radicals by leukocytes from periodontitis patients (Kimura et al., 1993). The contradictory findings from these studies might be related to the dynamics of the mechanisms during the pathogenesis of the disease, but they might also be explained by the different forms of periodontitis (Biasi et al., 1999).

The term oxidative stress is vague, similarly, antioxidants might affect many processes not directly related to free radical generation or action (Niki, 2016). This makes the interpretation of studies focusing on oxidative stress in periodontitis difficult. A systematic review of these clinical studies and animal experiments might, thus, be needed.

## Biomarkers of oxidative stress and pathogenesis of periodontitis

Surprisingly, many observational studies analyzing oxidative stress in patients with periodontitis had relatively consistent results with higher oxidative stress markers in either saliva or blood and/or decreased antioxidant status in comparison to controls. The summary table of the identified studies can be found in Table 1. One of the largest observational studies has shown that the antioxidant status in blood, analyzed as vitamin C, bilirubin, and calculated total antioxidant capacity was inversely associated with mild and severe periodontitis (Chapple et al., 2007b). The more severe periodontitis, the clearer the association. Additionally, in a subgroup of never-smokers the antioxidants seemed to protect against development of periodontitis. Others smaller studies confirmed these results. Total antioxidant capacity was lower in plasma or serum of patients with chronic periodontitis (Chapple et al., 2002; Brock et al., 2004; Konopka et al., 2007). Similarly, superoxide dismutase activity (Huang et al., 2014) along with catalase and glutathione peroxidase activity (Tonguç et al., 2011) as important contributor to the total antioxidant capacity was found to be lower in periodontitis. In line with these results from blood, in the majority of studies, the antioxidant status was lower also locally—in saliva. The total antioxidant status/potential/capacity is in general the ability of a tissue to resist artificially induced oxidative stress, but studies differ in the analytical approaches. Nevertheless, the local antioxidant capacity was found to be lower in saliva from patients with an aggressive form of periodontitis when compared to chronic periodontitis (Acquier et al., 2017). Patients with chronic periodontitis have lower antioxidant capacity than control patients (Zhang et al., 2015; Ahmadi-Motamayel et al., 2017). Although the total antioxidant capacity is not a specific marker of antioxidant power, uric acid, glutathione peroxidase (Miricescu et al., 2014), and reduced glutathione (Gumus et al., 2009) as specific antioxidants were reported to be significantly lower in saliva of patients with chronic or aggressive periodontitis. On contrary, activities of the major antioxidant enzymes were found to be higher in chronic periodontitis patients in all investigated samples, i.e., plasma, erythrocytes, and in the gingival tissue (Panjamurthy et al., 2005). Similarly, SOD2 and GPX1 genes were overexpressed in the gingiva of chronic periodontitis patients (Duarte et al., 2012). Lactoferrin, myeloperoxidase and interleukin 1 beta were all positively correlated with the clinical markers of periodontal damage (Wei et al., 2004). However, whether such associations of higher antioxidant and pro-inflammatory response are a consequence or cause of severe periodontitis cannot be judged only from observations.

**Table 1:** Observational studies analyzing oxidative stress in periodontitis.

**Study design**	**Sample size**	**Note**	**Sample type**	**Outcome**	**References**
Cross-sectional study	20 AgP patients 20 ChP patients 20 controls	–	Saliva	↑ ROS, TBARS with AgP; TRAP ↓ with AgP compared to ChP	Acquier et al., 2017
Case-control study (cross-sectional)	55 ChP patients 55 healthy controls	–	Serum Saliva	↓ TAC in serum and saliva; ↑ MDA increased in serum and saliva vs. controls	Ahmadi-Motamayel et al., 2017
Prospective study	33 PW with ChP 18 PW with gingivitis 21 PW controls 27 non-PW ChP 25 non-PW controls	Pregnancy	Serum GCF	↓ TAC, SOD in PW vs. non-PW and in ChP group compared to controls; ↑ TAC, SOD in third trimester in PW with ChP vs. PW with ChP in the first trimester	Akalin et al., 2009
Comparative study (cross-sectional)	20 T2DM patients with periodontitis 20 T2DM patients PH 20 SH patients with periodontitis	T2DM	Plasma	↓ plasma small molecule antioxidant capacity ↑ PC in T2DM with periodontitis vs. PH T2DM patients	Allen et al., 2011
Cross-sectional study	33 ChP patients 16 patients with gingivitis 37 healthy controls	–	Saliva	↑ TAC, 8-OHdG, MDA and activity of SOD, GPx in ChP vs. controls	Almerich-Silla et al., 2015
Observational study (cross-sectional)	19 non-T2DM patients 24 T2DM patients with good metabolic control 27 T2DM patients with poor metabolic control T2DM	T2DM	Saliva	↓ GPx, glutathione reductase in poor metabolic control T2DM patients; ↑ GSSG/GSH ratio in poor metabolic control T2DM	Arana et al., 2017
Case-control study	15 PH + normal weight 15 gingivitis + normal weight 15 ChP + normal weight 15 PH + obese 18 gingivitis + obese 15 ChP + obese	Obesity	GCF	↑ MDA, PC and ↓ TAC in ChP + obese group	Atabay et al., 2017
Cross-sectional study	32 ChP post-menopausal women 31 ChP pre-menopausal 25 PH post-menopausal women 26 PH pre-menopausal women	Menopause	Serum GCF	↓ TAC, SOD activity in post-menopausal women with ChP in serum and GCF	Baltacioglu et al., 2006
Cross-sectional study	35 AgP patients 33 ChP patients 30 PH controls	–	Serum Saliva	↑ MDA, TOS, OSI in periodontitis groups; ↓ TAC in periodontitis groups vs. controls (except serum MDA)	Baltacioglu et al., 2014
Cross-sectional study	23 ChP patients 19 PH controls	–	Saliva	↑ TBARS in ChP (men) ↓ TAC in ChP (women); trend toward ↓ DNA integrity in ChP	Banasová et al., 2015
Cross-sectional study	9 patients with periodontitis 9 healthy controls	–	Gingival tissue	↑ activities of MPO, GPx, glutathione S-transferase in periodontitis group; ↑ TBARS and GSSG levels increased vs. controls	Borges et al., 2007
Cross-sectional study	20 ChP patients 20 healthy controls	–	Serum Saliva GCF	↓ TAC in GCF and plasma in ChP vs. control; ↓ TAC in saliva in ChP vs. control	Brock et al., 2004
Cross-sectional study	32 ChP patients 32 PH control	–	Saliva Gingival tissue	↑ 8-OHdG and mtDNA deletions in ChP group vs. control	Canakci et al., 2009
Cross-sectional study	30 ChP patients 30 PH control	–	Blood Gingival tissue	↑ mtDNA deletion in ChP group vs. control	Canakci et al., 2006
Cross-sectional study	10 preeclampsia ChP 10 preeclampsia PH 10 normotensive ChP 10 normotensive PH	Preeclampsia	Serum Saliva GCF	↓ TAC in ChP women with preeclampsia (GCF, serum, saliva); ↓ SOD and GPx activities decreased in ChP women with preeclampsia (GCF and serum); ↑ MDA in ChP preeclamptic women (GCF, serum)	Canakci et al., 2007
Cross-sectional study	31 pre-menopausal 31 peri-menopausal 31 post-menopausal ChP women	Menopause	GCF Gingival tissue	↑ 8-OHdG in ChP post-menopausal women in GCF and gingival tissue	Chandra et al., 2017
Cross-sectional study	10 ChP patients 10 PH control	–	Plasma GCF	↓ TAC in GCF and plasma in ChP vs. control; ↓ GSH and GSSG in GCF in ChP	Chapple et al., 2002
Observational correlational study	11,480 participants; 1,567 with mild periodontitis 609 with severe periodontitis	–	Serum	↓ vitamin C, bilirubin and TAC (calculated) in mild or severe periodontitis	Chapple et al., 2007b
Cross-sectional study	17 periodontitis patients 20 healthy controls	–	Saliva	↓ TAC in periodontitis	Diab-Ladki et al., 2003
Cross-sectional study	12 SH and PH controls 15 SH and ChP patients 8 well-controlled T2DM patients with ChP 14 poor-controlled T2DM patients with ChP	T2DM	Gingival tissue	Peroxiredoxin 1 and GPX1 overexpressed in ChP; Peroxiredoxin 2 and SOD2 up-regulated especially in poor—controlled T2DM with ChP	Duarte et al., 2012
Cross-sectional study	20 ChP patients with RA 20 PH patients with RA 20 ChP patients SH 20 SH and PH controls	Rheumatoid arthritis	Serum GCF	↑ TOS in GCF of ChP and RA ChP groups (no difference for TOS and OSI in serum)	Esen et al., 2012
Cross-sectional study	18 ChP patients with hyperlipidemia 18 PH patients with hyperlipidemia 19 ChP patients SH 19 PH SH controls	Hyperlipidemia	Serum	↑ MDA and 8-OHdG in patients with ChP and hyperlipidemia	Fentoglu Ö et al., 2015
Cross-sectional study	24 ChP with depression 23 ChP without depression	Depression	Plasma	↑ nitric oxide metabolites, lipid peroxides, AOPP and TRAP in ChP with depression	Gomes et al., 2017
Cross-sectional study	16 T1DM with periodontitis 25 T2DM with periodontitis 24 SH with periodontitis	T1DM T2DM	Saliva	↓ GSH and ↓GSSG in the patients with T1DM	Gumus et al., 2009
Case-control study	115 P women (6 month postpartum follow-up) 72 non-P women	Pregnancy	Saliva	↑ 8-OHdG in PW; ↓ GPx decreased in PW; ↓ TBARS postpartum vs. non-pregnant women	Gümüş et al., 2015
Prospective study	218 P women 459 P women with mild periodontitis 114 P women with moderate-severe periodontitis	Pregnancy	Serum	↑ 8-isoprostane in PW with moderate-severe periodontitis	Hickman et al., 2011
Prospective study	50 ChP patients 50 healthy controls	–	Serum Saliva	↓ SOD in saliva and serum; ↑ prostaglandin E2, D2, prostaglandin F2α and TXB2, 5- hydroxyeicosatetraenoic acid, F2-isoprostanes; ↑ prostacyclin I2 and 13- hydroxyoctadecadienoic acid, 9- hydroxyoctadecadienoic acid	Huang et al., 2014
Cross-sectional Study	55 patients with DS 74 patients with mental retardation 88 healthy controls	Down Syndrome Mental retardation	Whole blood	↑ oxidative burst activity of blood (monocytes and granulocytes) in DS patients with decreased periodontal health	Khocht et al., 2014
Cross-sectional study	26 AgP patients 30 ChP patients 25 healthy controls	–	Gingival blood Peripheral blood	↑ 8-OHdG in gingival blood of ChP and AgP patients; ↓ TAC in gingival blood of ChP patients; ↓ TAC in peripheral blood of both groups	Konopka et al., 2007
Cross-sectional study	1,258 old men	Old age	Serum	↓ beta-cryptoxanthin and beta-carotene with decreased periodontal health of old men	Linden et al., 2009
Cross-sectional study	356 periodontitis patients 207 PH controls	–	Plasma	↑ reactive oxygen metabolites and shorter leukocyte telomere length in ChP	Masi et al., 2011
Cross-sectional Correlational study	20 ChP patients 20 PH controls	–	Saliva	↑ 8-OHdG, MDA in ChP; ↓ uric acid, GPx activities and TAC in ChP (correlation with bone loss markers)	Miricescu et al., 2014
Cross-sectional study	10 T2DM patients PH 8 SH controls	T2DM	Periodontal tissue	↑ MDA, ↓ GSH in periodontal tissue of T2DM	Monea et al., 2014
Cross-sectional study	24 ChP patients with ACS 24 patients PH with ACS 24 ChP patients without ACS 24 controls PH without ACS	Acute coronary syndrome	Saliva	↑ 8-OHdG, MDA, and PC in patients (correlation with periodontal and cardiovascular markers)	Nguyen et al., 2016
Cross-sectional study	25 ChP patients 25 healthy controls	–	Plasma Gingival tissue Erythrocytes	↑ TBARS in ChP; ↑ SOD, CAT, GPx activities in ChP; ↓ vitamins E, C and GSH in ChP	Panjamurthy et al., 2005
Cross-sectional study	29 ChP patients 20 healthy controls	–	Saliva	↑ 8-OHdG in ChP (correlation with *P. gingivalis*)	Sawamoto et al., 2005
Cross-sectional cohort study	46 severe periodontitis patients 37 moderate periodontitis patients 46 mild periodontitis and healthy	–	Saliva	↑ PC increased in severe periodontitis; ↓ urate and FRAP in severe periodontitis	Sculley and Langley-Evans, 2003
Cross-sectional study	20 ChP patients with RA 20 PH patients with RA 20 ChP patients without RA 20 PH SH controls	Rheumatoid arthritis	Serum	↑ OSI and prolidase in ChP patients with RA	Sezer et al., 2013
Cross-sectional study	20 ChP patients with PS 20 PH patients with PS 20 ChP patients with PsA 20 PH patients with PsA 20 ChP SH patients 20 PH SH controls	Psoriasis Psoriatic arthritis	Serum	↑ OSI (irrespective of periodontitis) in patients groups; (PS and PsA showed no effect on clinical parameters in ChP patients)	Sezer et al., 2016
Cross-sectional study	4,717 participants	Diabetes mellitus Hypertension	Serum	Periodontitis with highest 8-isoprostane quartile associated with ↑ CRP	Singer et al., 2015
Cross-sectional correlational study	29 severe periodontitis 77 moderate periodontitis 96 mild periodontitis and healthy	Diabetes mellitus Hypertension	Serum	↑ ROM in patients with worst periodontal status	Tamaki et al., 2014b
Cross-sectional study	25 severe periodontitis 43 moderate periodontitis 92 mild periodontitis and healthy	Diabetes mellitus Hypertension	Saliva	Superoxide and hydroxyl radical scavenging activities associated with periodontitis	Tamaki et al., 2015
Cross-sectional study	39 patients with periodontitis	–	Saliva	↓ antioxidant concentrations related to periodontal status	Tartaglia et al., 2017
Cross-sectional study	23 smokers with ChP 23 former smokers with ChP 19 non-smokers with ChP 20 PH non-smokers controls	Smoking	Serum Gingival tissue	↑ MDA in serum and gingival tissue in smoking ChP patients groups; ↓ SOD, CAT and GPx in ChP groups	Tonguç et al., 2011
Cross-sectional study	82 children	–	Saliva	TBARS in correlation to periodontal status; TAC related to periodontal status and oral hygiene; AOPP related to caries in children	Tothova et al., 2013
Cross-sectional study	30 ChP patients with T2DM 30 ChP SH patients 30 PH patients with T2DM 30 PH SH controls	T2DM	Plasma Saliva Red blood cell lysate	↑ MDA in ChP patients irrespective of T2DM	Trivedi et al., 2014
Cross-sectional study	100 ChP patients 50 healthy controls	–	Saliva	↑ 8-OHdG and human neutrophil elastase/alpha1-proteinase inhibitor in ChP	Villa-Correa et al., 2015
Cross-sectional study	19 patients with periodontitis 8 healthy controls	-	GCF	↑ GPx, lactoferrin, myeloperoxidase and IL-1beta in periodontal tissues (correlation with clinical periodontal markers)	Wei et al., 2004
Cross-sectional study	31 smokers 90 non-smokers	Smoking	Saliva	↑ 8-epi-PGF(2alpha) with periodontal status and smoking; Smoking ↑ TXB(2) and PGF(2alphas) and ↓ 6-oxo-PGF(1alpha)	Wolfram et al., 2006
Cross-sectional study	58 ChP patients 42 AgP patients 60 healthy controls	–	Saliva Buccal mucosa	↑ micronuclei and nuclear abnormalities, as well as 8-OHdG in both periodontitis groups	Zamora-Perez et al., 2015
Cross-sectional study	45 severe periodontitis patients 37 healthy controls	–	Saliva	↓ TAC in periodontitis (TOS no difference)	Zhang et al., 2015

*ChP, chronic periodontitis; AgP, aggressive periodontitis; SH, systematically healthy; PH, periodontal healthy; T1DM, type 1 diabetes mellitus; T2DM, type 2 diabetes mellitus; PS, psoriasis; PsA, psoriatic arthritis; DS, Down syndrome; PW, pregnant woman; RA, Rheumatoid arthritis; ACS, Acute coronary syndrome; GCF, gingival crevicular fluid; TAC, total antioxidant capacity; TBARS, thiobarbituric acid reacting substances; 8-HOdG, 8-hydroxydeoxyguanosine; GPx, glutathione peroxidase; GSH, reduced glutathione; GSSG, oxidized glutathione; PC, protein carbonyls; MDA, malondialdehyde; TOS, total oxidant status; SOD, superoxid dismutase; OSI, oxidative stress index; IL-1beta, interleukin 1beta; ROM, reactive oxygen metabolites; ROS, reactive oxygen species; FRAP, ferric reducing antioxidant power; 4-HNE, 4-hydroxy-2-nonenal; CAT, catalase; CRP, C-reactive protein; AOPP, advanced oxidation protein products; TRAP, total radical-trapping antioxidant potential; MPO, myeloperoxidase; mtDNA, mitochondrial DNA*.

Regarding the analyzed markers of oxidative stress, a comparison of the published studies is complicated, if not impossible due to the huge variability of measured markers. Malondialdehyde, 8-hydroxydeoxyguanosine (Konopka et al., 2007; Almerich-Silla et al., 2015), protein carbonyls (Nguyen et al., 2016; Atabay et al., 2017), thiobarbituric acid reacting substances (Borges et al., 2007), nitric oxide, advanced oxidation protein products, lipid peroxidation products (Gomes et al., 2017), 8-isprostanes (Hickman et al., 2011) were all higher in patients with periodontitis. The most commonly measured markers of oxidative stress seem to be malondialdehyde and thiobarbituric acid reacting substances pointing toward oxidative damage of lipids, especially, lipid membranes. Lipid peroxidation was higher in saliva (Tothova et al., 2013), serum (Tonguç et al., 2011), and in the gingival tissue (Panjamurthy et al., 2005) of patients with chronic or aggressive periodontitis. Correlational studies confirmed a positive association of these markers with periodontal status scores (Chapple et al., 2007b; Tamaki et al., 2014b). The less commonly measured markers related to oxidative damage as mitochondrial DNA (Canakci et al., 2006), micronuclei and nuclear abnormalities (Zamora-Perez et al., 2015), as well as a leukocyte telomere length shortening (Masi et al., 2011) were all higher in periodontitis as well.

Oxidative stress was found to be involved in the pathogenesis of many diseases besides periodontitis. Virtually, almost all inflammatory diseases lead to increased oxidative stress. This in turn can trigger more damage to the tissues, not excluding the gingival tissue and, thus, worsening periodontitis. There are several studies describing oxidative stress in systemic diseases with regard to the periodontal status. Nguyen et al. (2016) investigated patients with the acute coronary syndrome with or without chronic periodontitis, patients with periodontitis and healthy controls. Lipid, protein, and DNA oxidation markers were higher in periodontitis than in the control group (Nguyen et al., 2016). Another study with rheumatoid arthritis and chronic periodontitis confirmed higher oxidative stress markers in plasma and lower antioxidant capacity in both groups when compared to healthy control. However, if both comorbidities were present, there was no further enhancement of oxidative stress (Sezer et al., 2013).

Taken together, observational cross-sectional studies confirmed the association of oxidative stress and periodontitis. Higher oxidative stress and lower antioxidant status can be detected in plasma, saliva as well as in the gingival crevicular fluid of patients with various clinical forms of periodontitis. These findings support the use of body fluids, but especially the non-invasive diagnostic fluid saliva, as suitable sample types for diagnostics or monitoring the course of periodontitis. Current data do not support the use of a single oxidative stress marker. It is likely that a set of markers covering both, oxidative damage and antioxidative status, will be needed. The low specificity of oxidative stress markers calls for caution when interpreting the results even if more than one marker is used. The inter-individual and intra-individual variability of the analyzed markers is very high. This prevents their use at the level of individual diagnostics. The overview of the published observational studies shows the enormous heterogeneity of the patient populations as well as the used analytical tools. In a meta-analysis focusing on systematic oxidative stress, it was shown that higher malondialdehyde and nitric oxide as well as lower total antioxidant capacity of plasma/serum characterize patients with periodontitis in comparison to controls (Liu et al., 2014). Based on our overview, taken into account systemic and local oral biomarkers of oxidative stress, it is clear that there is a need for both, the use of a wider palette of markers to analyze oxidative stress and its causes in more detail, and the introduction of new biomarkers with a better sensitivity/specificity profile in specific subgroups of patients. Of special importance is the small sample size in most studies. A collaborative effort with a multi-center recruitment of patients and a standardized consensus protocol in the pre-analytical and analytical phase is highly needed.

## Oxidative stress and treatment of periodontitis

The implication of oxidative stress in the pathogenesis of cardiovascular diseases and cancer as the major causes of death in the combination with the widespread availability of dietary antioxidants started a hype that was supported by the hypothesis that aging is caused by oxidative stress (Harman, 1956; Finkel and Holbrook, 2000). However, the hype was quickly over as large clinical studies revealed that antioxidants or at least antioxidant vitamins were not able to prevent any of the diseases of aging (Coulter et al., 2006; Sesso et al., 2008; Myung et al., 2013). Some studies even revealed a slight but increased risk in patients taking antioxidants in preeclampsia (Rumbold et al., 2006) and lung cancer (Alpha-Tocopherol, 1994). One meta-analysis showed that taking antioxidant may even increase the all-cause mortality by 5% (Bjelakovic et al., 2007). This might be related to the terminological and mechanistic confusion about antioxidants, which might have a very indirect effect on the production or effects of reactive oxygen species. In an era of evidence-based medicine the clear conclusion is that antioxidant dietary supplements should undergo clinical evaluation before marketing similarly to other medicinal drugs (Bjelakovic et al., 2012). Recent mechanistic experiments shed light on the details how antioxidants may stimulate tumor growth (Sayin et al., 2014) or increase the risk of metastasis (Piskounova et al., 2015). Antioxidants are highly variable in their mechanism and structure. Diseases and patients vary even more. So, negative results from oncology or cardiology should not be generalized to other diseases including periodontitis. It is very likely that the role of oxidative stress changes during disease progression and, thus, the potential antioxidant treatment affecting not only oxidative damage but also the inflammatory process might have different affects at various stages of the complex pathogenesis. The issue of antioxidants that mostly do not affect free radicals and their action *in vivo*, but rather interfere with variable cellular signaling pathways has been reviewed in a recently published paper (Azzi, 2017).

Many published studies have analyzed the effects of periodontitis treatment on oxidative stress (Table 2). Studies of special interest are those with covariates or comorbidities that were taken into account. A trial by Guentsch et al. (2008) examined healthy subjects and patients with periodontitis further divided into smokers and non-smokers. While smokers with periodontitis displayed highest malondialdehyde concentration and highest glutathione peroxidase activity along with lowest total antioxidant capacity in saliva. The non-surxgical treatment helped to normalize the values regardless of the smoking status (Guentsch et al., 2008). Several other trials confirmed the consistency of these findings (Chapple et al., 2007a; Abou Sulaiman and Shehadeh, 2010; Hendek et al., 2015). On the other hand, a severe systemic condition such as type 2 diabetes may lead to inefficiency of non-surgical therapies to improve the periodontal status or oxidative stress (Koromantzos et al., 2012). In general, most of the studies clearly show an improvement of oxidative damage after standard treatment of periodontitis. The studies, however, vary greatly regarding sample types, markers measured, and most cohorts were very small and highly variable. Thus, making relevant conclusions or recommendations for the clinical dentistry is difficult, if not impossible. Future research efforts should focus on the lack of uniformity and standardization as well as on the issue of the low informative value of small patient cohorts.

**Table 2:** Interventional studies analyzing the effect of periodontitis on oxidative stress.

**Treatment**	**Treatment duration**	**Sample size**	**Comorbidity**	**Sample type**	**Results**	**References**
NST with vitamin C (2,000 mg/day)	1 month	30 ChP patients 30 healthy controls	None	Plasma	↓ TAC in ChP patients; NST lead to ↑ TAC in ChP patients; No effect of vitamin C	Abou Sulaiman and Shehadeh, 2010
NST with lycopene (8 mg/day)	2 month	20 ChP patients	None	Serum	↓ MDA and clinical parameters after therapy	Ambati et al., 2017
Oral hygiene education with insulin treatment for T1DM	3 months	32 T1DM patients at diagnosis 18 SH children with gingivitis 18 SH and PH children	T1DM	Serum Saliva GCF	↑ serum, salivary and GCF OSI in T1DM group; ↓ after treatment	Aral et al., 2017
NST with lycopene (8 mg/day)	–	42 ChP patients	None	Plasma Saliva	↑ IL-1ss, UA and clinical parameters after treatment	Arora et al., 2013
NST	14 days	13 ChP patients with FMF 15 ChP SH patients 14 PH patients with FMF 15 PH and SH controls	Familial Mediterranean fever	Serum GCF	↓ periodontal clinical markers after treatment; ↓ TOS in GCF in FMF patients with ChP; no difference in serum TOS and OSI	Bostanci et al., 2014
ST with antioxidant gel (2mg lycopene)	1 week	31 ChP patients	None	GCF	↓ 8-OHdG after treatment, as well as periodontal clinical markers	Chandra et al., 2013
NST	2 months	35 ChP patients 32 healthy controls	None	Plasma GCF	↓ TAC in GCF in ChP; ↑ after treatment; (plasma TAC no difference at baseline between groups, no change after treatment)	Chapple et al., 2007a
NST	4 h	145 ChP patients (14 with therapy) 56 healthy controls	None	Serum	Patients with severe periodontitis ↑ROM ↓ TAC; ↑ ROM after treatment	D'Aiuto et al., 2010
NST with fluorescence-controlled Er:YAG laser radiation	Laser therapy applied 1 day after NST	30 ChP patients	None	GCF	no difference between with/without laser treatment in TAC and clinical parameters; IL-1beta and ↑ TNF-alpha after NST only; ↓ after NST with laser	Dominguez et al., 2010
NST	–	30 ChP patients 30 healthy controls	Smokers/non-smokers	Serum Saliva	↑ MDA in smoking ChP patients; ↑ GPx in ChP groups; ↓ TAC in ChP groups; ↓ MDA and GPx after therapy	Guentsch et al., 2008
NST	14 days	47 (24 / 23) ChP patients 46 (23/23) healthy controls	Smokers/non-smokers	Serum Saliva GCF	↑ salivary 8-OHdG and GPx in ChP; ↑ 4-HNE in GCF in ChP smokers; ↓ 8-OHdG after therapy in saliva and GCF	Hendek et al., 2015
NST	–	7 ChP patients 7 healthy controls	None	Saliva	↑ TAC and ↓ SOD activity in ChP; ↓ TAC, SOD activity immediately after NST	Kim et al., 2010
NST	–	60 moderate to severe periodontitis patients with T2DM	T2DM	Serum	No effect of NST on d-8-iso, MMP-2, MMP-9 and hsCRP	Koromantzos et al., 2012
NST; NST with antioxidants (6 mg/day; lycopene, zinc, and selenium) or antioxidants only	3 doses in 2 weeks	30 ChP patients 30 gingivitis patients 10 healthy controls	None	Saliva	↓ UA in ChP patients; antioxidant treatment ↑ UA	Mathur et al., 2013
Tai Chi (5 days a week, 60 min)	6 months	71 sedentary patients with periodontitis	Old age	Saliva	↑ TAC, SOD after therapy	Mendoza-Núñez et al., 2014
NST and surgical treatment	Surgical treatment 6 weeks after NST	12 AgP patients	None	Serum Plasma	Periodontitis severity associated with LDL concentrations; No changes in lipid profile after treatment; No difference in GSH and lipid hyperoxide after therapy	Nibali et al., 2015
NST or oral hygiene instructions	2 visits within 7 days	42 ChP patients 21 healthy controls	None	Saliva	NST lead to ↑ TAC, ALB, UA, GPx and ↓ SOD; (no effect of oral hygiene instructed therapy was found)	Novakovic et al., 2014
NST	–	25 severe ChP patients 26 healthy controls	None	Serum Saliva	Salivary 8-OHdG ↑ before treatment in ChP group; ↓ after NST ↑ salivary MDA and serum 4-HNE in ChP patients; NST had no effect	Onder et al., 2017
NST with Coenzyme Q10; NST with tea tree oil gel or placebo	7 days	15 ChP patients (moderate to severe)	None	-	Both antioxidant treatment procedures effective in ↓ clinical markers of ChP (PI, GI, PPD and CAL)	Raut and Sethi, 2016
NST and oral hygiene instructions	2–4 months	29 ChP patients 20 healthy controls	None	Saliva	↑ 8-OHdG in ChP patients before therapy; ↓ 8-OHdG and *P. gingivalis* after treatment, as well as periodontal clinical markers	Sawamoto et al., 2005
NST	–	8 ChP patients 8 healthy controls	None	Platelet suspension	↓ periodontal clinical parameters and CRP after therapy, as well as ↑ cGMP and SOD activity	Siqueira et al., 2013
Surgical treatment with taurine (500 mg/day)	15 days	10 ChP patients	None	Plasma Gingival tissue	↓ TBARS, GPx in plasma and gingival tissue; ↑ GSH and ↓ periodontal clinical parameters after therapy	Sree and Sethupathy, 2014
NST	–	78 ChP patients 17 healthy controls	None	Saliva	↑ 8-OHdG in ChP patients before therapy; ↓ 8-OHdG and periodontal clinical markers after therapy	Takane et al., 2002
NST	–	22 ChP patients 22 healthy controls	None	Plasma	↑ oxidative index, oxidized LDL and CRP in ChP; ↓ of these parameters after treatment	Tamaki et al., 2011
NST	–	25 ChP patients 25 patients with gingivitis 25 healthy controls	None	Serum	↓ serum TAC and CAT in both groups of patients; TAC ↑ in ChP patients after therapy (no treatment effect on CAT)	Thomas et al., 2014
NST	4 visits within 14 days	30 (15/15) ChP patients 10 healthy controls	Smokers/non-smokers	GCF	↑ IL-1beta in ChP patients; ↓ IL-1beta after NST irrespective of smoking; (no difference in TAC and TOS before or after treatment between groups)	Toker et al., 2012
NST	2 weeks	25 ChP patients with MS 25 ChP SH patients	Metabolic syndrome	Serum Saliva	TOS and OSI showed no difference between groups in serum after therapy; ↑ TAC of MS ChP patients before treatment, but ↓ after therapy in serum; ↓ OSI and ↑ TAC in both groups after treatment in saliva	Torumtay et al., 2016
NST	Once per week/1 month	22 ChP patients	None	Saliva	↑ SOD in patients with low dental visits after NST; ↑ TAC in patients with regular dental visits after therapy	Yang et al., 2014
NST with dietary intervention	3 visits/6 months	37 ChP patients (19 without intervention; 18 with intervention)	None	Plasma Saliva	↑ TAC after dietary intervention in plasma; no differences in periodontal clinical parameters after dietary intervention	Zare Javid et al., 2014

*ChP, chronic periodontitis; SH, systematically healthy; PH, periodontal healthy; NST, non-surgical treatment; ST, surgical treatment; T1DM, type 1 diabetes mellitus; T2DM, type 2 diabetes mellitus; FMF, familial Mediterranean fever; MS, metabolic syndrome; GCF, gingival crevicular fluid; TAC, total antioxidant capacity; TBARS, thiobarbituric acid reacting substances; 8-HOdG, 8-hydroxydeoxyguanosine; GPx, glutathione peroxidase; GSH, reduced glutathione; MDA, malondialdehyde; TOS, total oxidant status; SOD, superoxid dismutase; OSI, oxidative stress index; IL-1ss, salivary interleukin 1beta; ROM, reactive oxygen metabolites; 4-HNE, 4-hydroxy-2-nonenal; hsCRP, high-sensitivity C-reactive protein; d-8-iso, d−8-iso prostaglandin F2a; MMP-2, matrix metalloproteinase 2; MMP-9, matrix metalloproteinase 9; LDL, low density lipoprotein; ALB, albumin; UA, uric acid; CAT, catalase; CRP, C-reactive protein; PI, plaque index; GI, gingival bleeding index; PPD, probing pocket depth; CAL, clinical attachment level; TNF-alpha, tumor necrosis factor alpha; cGMP, L-arginine-nitric oxide (NO)-cyclic guanosine monophosphate*.

Although the causality of the association between oxidative stress and periodontitis is everything but clear, some clinical studies already tested antioxidants in periodontitis. One small, placebo controlled, randomized, and double-blind study showed, that single application of lycopene gel to periodontitis patients improved clinical attachment and decreased oxidative stress in gingival crevicular fluid (Chandra et al., 2013). Nevertheless, a published systematic review on antioxidant treatment of periodontitis revealed that a consistent effect in randomized clinical trials was found only for lipophile antioxidants such as lycopene and vitamin E, but not for hydrophile antioxidants such as vitamin C (Muniz et al., 2015). This might be related to the vulnerability of lipids to oxidative damage, but also to mitochondria as the site of effect of some antioxidants. Interestingly, antioxidants targeting directly mitochondria have been shown to be effective in decreasing inflammatory activity and organ damage in animal model of sepsis (Lowes et al., 2013).

## Animal experiments

The high number of observational and interventional studies analyzing the association between oxidative stress and periodontitis indicates that there are many open questions that cannot be answered by more and more clinical studies. Many of the questions need controlled conditions in experiments. The number of animal experiments analyzing the role oxidative stress in periodontitis is small, but it increases. Periodontitis is mostly induced by ligature placement around the first molars of the animals or local injection of periodontal pathogens or their toxins (Genco et al., 1998; Fine, 2009; Oz and Puleo, 2011). In such a rat model, it was shown that periodontitis leads to an increased production of reactive oxygen species and markers of oxidative damage (Ekuni et al., 2010). In addition, oxidative stress induced by periodontitis seems to be associated with the dynamics and severity of the periodontal inflammation (Bosca et al., 2016).

Some animal experiments focus on the distant effects of periodontitis that seem to be mediated by oxidative stress. It was shown that mitochondrial DNA is oxidatively modified in the liver, kidney, heart, and brain of rats with induced periodontitis (Tomofuji et al., 2011). Another similar experiment by the same group has shown that periodontitis might worsen ethanol-induced liver damage (Tomofuji et al., 2008). The oxidative damage to the heart, but also endothelial dysfunction and resulting atherosclerosis induced by experimental periodontitis can be prevented by antioxidant treatment (Ekuni et al., 2009; Ozdem et al., 2017; Saito et al., 2017). A majority of the published experiments focus on the use of antioxidants such as vitamin C (Tomofuji et al., 2009b), N-acetylcysteine (Toker et al., 2009), or resveratrol (Tamaki et al., 2014a), but also drugs with an antioxidant activity beyond their main mechanism of action (de Araujo Junior et al., 2013; Culic et al., 2014; Oktay et al., 2015). Dietary interventions have also been investigated—high cholesterol diet seems to worsen periodontitis (Tomofuji et al., 2006). On contrary, the phytoestrogen genistein and cacao-enriched diet was shown to be protective against periodontal damage and oxidative stress induced by periodontitis in mice and rats, respectively (Tomofuji et al., 2009a; Bhattarai et al., 2017). The most promising candidate drug at least according to animal experiments is melatonin. This amphiphile molecule has an optimal distribution in the tissues and can, thus, reach the periodontal tissues even after systemic administration (Köse et al., 2017). Of special clinical relevance is the induction of periodontitis in diabetic rats since periodontitis is a common complication of diabetes in humans. Melatonin was able to prevent alveolar bone loss also in this experimental model (Kose et al., 2016). However, regarding the mechanism of action, it is not clear whether the protective effect of melatonin is due to its direct antioxidant characteristics or due to its immunomodulatory effects that might reduce oxidative stress indirectly (Kara et al., 2013). This uncertainty is not specific for melatonin. Any antioxidant might affect the immune response and, thus, have anti-inflammatory properties. Beyond established systemic antioxidants novel approaches with a local periodontal application are tested (Saita et al., 2016). Experimental tools such as genetically engineered mice that produce luciferase under the regulation of transcription factors related to oxidative stress and antioxidant response have been developed and might greatly improve our understanding of the role of oxidative stress in periodontitis (Kataoka et al., 2016). The new treatment options together with new and improved models could be very helpful in the fight against this widespread and serious disease.

## Conclusion

The role of oxidative stress in periodontitis is not clear despite decades of research. Numerous studies have been published showing the potential of oxidative stress markers for screening, diagnosis or monitoring of the disease, but none is in routine clinical use. Similarly, animal experiments, as well as most of the interventional studies in patients, indicate that antioxidant treatment should be effective in the therapy of periodontitis, but no such treatment has been approved. The lack of translation could be either due to the lack of strong evidence for the clinical usefulness or due to obstacles in the application of the results including the low or absent commercial interest from major stakeholders. From a research perspective, an important issue is the lack of specificity—both, in diagnostics and treatment. Not even the source of free radical production is clear. While some studies point toward neutrophils (Katsuragi et al., 2003), others show that bacteria actively producing reactive oxygen species might contribute to oxidative stress in periodontitis (Huycke et al., 2002; Vlkova and Celec, 2009). It is of crucial importance that the number of conducted animal experiments in this field is increasing, especially of those focusing on the dynamics of oxidative stress during disease progression. The antioxidant treatment might be effective only in a subset of patients during a specific stage of periodontitis. The shift from pure observations to interventions and animal experiments that can be followed in the published literature in the recent years is highly positive and should bring this field of research closer to true clinical applications despite chronic lack of financial support and human resources.

## Author contributions

L'T has analyzed the data from the literature, prepared tables, and drafted the manuscript; PC has designed the review, conducted the literature search, and drafted the manuscript.

### Conflict of interest statement

The authors declare that the research was conducted in the absence of any commercial or financial relationships that could be construed as a potential conflict of interest.
